# Bacillus Coli Infections of the Urinary Tract

**Published:** 1912-02

**Authors:** 


Bacillus Coli Infections of the Urinary Tract: Reginald M. Rawls, of New York, *Medical Record,* October 7, 1911. In a certain percentage of adults, even in health, the bacillus coli is taken up by the lymphatics and blood vessels of the intestines and deposited by the blood current in the kidneys. The number and virulence of these microorganisms depend on the intestinal disturbance and the particular strain present.

The greatest predisposing cause of bacillus coli infection of the urinary tract is anything that interferes with a free secretion of urine or causes any back pressure in the urinary tract.

The earliest symptoms of bacillus coli infection of the urinary tract are apt to be mistaken for those of a mild cystitis, whereas the real site of infection is most often higher up in the urinary tract and of more serious pathologic import.

With greater care in diagnosis we will find that bacillus coli infections of the urinary tract are likely to be mistaken for other acute conditions, such as appendicitis, malaria, intestinal indigestion, toxemia of pregnancy, ether nephritis and acute and chronic nephritis. This is especially true of post-operative gynecological cases.

